# Modulation of Heat Shock Proteins Levels in Health and Disease: An Integrated Perspective in Diagnostics and Therapy

**DOI:** 10.3390/cells14130979

**Published:** 2025-06-25

**Authors:** Elena Mikhailova, Alexandra Sokolenko, Stephanie E. Combs, Maxim Shevtsov

**Affiliations:** 1 Personalized Medicine Centre, Almazov National Medical Research Centre, 2 Akkuratova Str., 197341 Saint Petersburg, Russia; mikhailovaer@yandex.ru; 2 Department of Molecular Biotechnology, Saint-Petersburg State Institute of Technology, 190013 Saint Petersburg, Russia; alex_sokol_03@mail.ru; 3 Department of Radiation Oncology, Technishe Universität München (TUM), Klinikum Rechts der Isar, Ismaninger Str. 22, 81675 Munich, Germany; stephanie.combs@tum.de; 4 Laboratory of Biomedical Nanotechnologies, Institute of Cytology of the Russian Academy of Sciences (RAS), 194064 Saint Petersburg, Russia

**Keywords:** heat shock proteins, chaperones, HSP70, HSP90, HSF, biomarker, inhibitors

## Abstract

Heat shock proteins belong to a highly conserved family of chaperone proteins, and in addition to their participation in the regulation of cellular proteostasis (folding of polypeptides and proteins, disaggregation of incorrectly folded peptides, and participation in autophagy processes), also play a significant immunomodulatory role in both innate and adaptive immunity. Changes in the HSP level, both downwards (e.g., in neurodegenerative diseases) and upwards (e.g., autoimmune, oncological diseases), underlie the pathogenesis of many somatic and oncological pathologies. In this review, we consider the main physiological mechanisms of HSP level regulation and also analyze pharmacological, genetically engineered methods of modulating the chaperone level, citing the advantages and disadvantages of a particular method of influence. In conclusion, modulation of the HSP level, according to numerous preclinical studies, can have a significant impact on the course of various pathological conditions, which, in turn, can be used to develop new therapeutic approaches, when the effect on the level of chaperones can be used as monotherapy or as an adjuvant method of action.

## 1. Introduction

Heat shock proteins (HSPs) are a widely distributed and highly conserved family of proteins in both prokaryotic and eukaryotic organisms. They play a crucial role in maintaining cellular proteostasis and protecting cells from metabolic stress. These proteins were named following the discovery by Tissieres et al. in 1974 that in response to an increase in ambient temperature (heat shock), the synthesis of a special group of proteins is activated in Drosophila larvae [1]. Later, it was found that the synthesis of these proteins is induced by a wide range of harmful stimuli, such as hypoxia and ischemia, as well as damaging chemical agents, such as poisons and toxic heavy metals [2,3,4,5,6].

They function as molecular chaperones within cells and form an integrated network that participates in various processes, including the folding of newly synthesized polypeptides, the refolding of metastable proteins, the assembly of protein complexes, the dissociation of aggregates, and the degradation of misfiled proteins. In addition to these chaperone functions, HSPs also play a crucial role in cell signaling, cell cycle regulation, and apoptosis. Impaired HSP functioning has been linked to various diseases, including cancer, neurodegenerative disorders, and others.

HSPs are classified into families based on their molecular weight, and to date, six major HSP families have been identified: large HSPs (100 kDa and above), HSP90, HSP70, HSP60, HSP40 (J-proteins), and small HSPs (proteins with molecular weight ranging from 12 to 43 kDa). Large HSPs perform a protective function, safeguarding the organism under stress conditions, and participate in the proper folding and disaggregation of proteins. HSP90 forms a complex with several auxiliary co-chaperone proteins and interacts with steroid hormone receptors, ensuring efficient hormone binding to the receptors and subsequent transport of the hormone receptor complex into the nucleus. Additionally, HSP90 facilitates the transport of several types of protein kinases to their functional sites. HSP60 is involved in the folding of complex multidomain proteins as well as in the ATP-dependent correction of errors in the structure of partially denatured proteins. HSP40 contains a J-domain which interacts with HSP70 and regulates its ATPase activity. It also binds to unfolded polypeptide chains and prevents their aggregation. Small heat shock proteins, or HSPs, are special helpers in our cells that do not need energy to work. They keep proteins from clumping together when things become tough, which is really important for cell health. They also help hold the cell’s shape and manage cell death to keep everything balanced. A recent study by Hu and others in 2022 showed that these small HSPs can come together to form bigger groups and are found throughout the cell—in the cytosol, mitochondria, and nucleus. Their main job is to grab onto proteins that are misfolded or not quite right, stopping them from clumping up and helping to keep everything in check [7].

The 70 kDa protein family consists of several related proteins that are typically found in various intracellular localizations [8,9]. In mammalian cells, these include HSC70 (cytoplasm/nucleus) [10,11]; HSP70 (cytoplasm/nucleus) [12,13,14,15,16]; Grp78/Bip (endoplasmic reticulum lumen) [17,18]; Grp75/mtp70 (mitochondrial matrix) [19,20]; and Ly- HSP70 (lysosomes) [21]. HSC70 and HSP70 are of particular interest because they not only localize in the same cellular compartments, but also exhibit a high degree of homology (≈95%) and demonstrate similar biological properties [22]. Moreover, HSC70 is constitutively synthesized in mammalian cells and is only moderately induced by stress, whereas HSP70 is generally undetectable under normal conditions but is strongly induced in cells experiencing stress. These proteins are often referred to as the constitutive and inducible forms of the 70 kDa HSP proteins.

The induction of heat shock proteins (HSPs) in response to various stressors depends on the activation of specific transcription factors, such as heat shock factor (HSF). These proteins bind to specific sequences in the promoters of HSP genes. Four members of the HSF family have been identified: HSF1, HSF2, HSF3, and HSF4. HSF1 and HSF3 are involved in regulating HSP expression in response to heat stress, while HSF2 and HSF4 are involved in non-stress-related processes, such as immune responses and cellular differentiation. However, HSP regulation is not solely dependent on HSF. Other transcription factors can interact with HSF and modify its activity, leading to changes in HSP gene expression in response to non-heat stress stimuli [23].

## 2. Molecular Regulation of HSPs

### 2.1. The Regulation of HSP Transcription by the HSF Family

#### 2.1.1. HSF1

Under non-stressful conditions, HSF1 exists mainly as an inactive monomer located throughout the nucleus and cytoplasm. In its monomeric form, HSF1 activation is suppressed by interaction with chaperones such as the heat shock proteins Hsp70 and Hsp90, and TRiC/CCT. In the case of proteotoxic stress, such as heat shock, these chaperones are released from HSF1 to perform their protein folding functions; at the same time, HSF1 export to the cytoplasm is inhibited. These actions allow HSF1 to trimerize and accumulate in the nucleus, binding to the heat shock elements (HSEs) region located either in the promoter regions of various HSP genes or in the intronic regions of non-HSP genes, which stimulates transcription of target genes [24].

The pivotal role of HSF1 was confirmed by the data showing that cells deprived of this crucial factor showed defects in HSP induction following exposure to heat shock and were prone to apoptotic death after exposure to heat stress. In addition, mice lacking HSF1 also had elevated levels of tumor necrosis factor α (TNF-α), which led to increased mortality after exposure to endotoxin and inflammation [25].

HSF1 is negatively regulated by HSP70 and HSP90, indicating the existence of a negative feedback mechanism for the regulation of HSP70 and HSP90 genes in response to heat shock. HSF1 is known to undergo post-translational modification through various processes including phosphorylation, acetylation, and sumoylation. Both phosphorylation and sumoylation play roles in regulating the transactivation potential of HSF1. Additionally, it has been demonstrated that p300 acetylates HSF1, and the deacetylation process mediated by NAD+-dependent sirtuin (SIRT1) reduces the heat shock response by preventing HSF1 acetylation and DNA binding [25].

Kinases responsible for phosphorylation of HSF1 at several serine sites include glycogen synthase kinase 3β (GSK3β) and c-jun N-terminal kinase (JNK). It has been shown that the cytokine interleukin 6 (IL-6) suppresses HSF1 by reducing the activity of GSKb. However, it is also known that HSF1 phosphorylation plays a positive role in stress-induced activation of Hsp gene expression. The exact mechanism of this effect has not been fully elucidated, although the CK2 protein kinase appears to be involved in enhancing transcriptional activity and DNA degradation. HSF1 binding occurs through the phosphorylation of threonine 142 residues. It is assumed that activation may also include dephosphorylation of HSF1 [25].

#### 2.1.2. HSF2

HSF2 is currently described as a factor involved in the regulation of various HSPs under non-stressful conditions. HSF2 exists as two isoforms, HSF2a and HSF2b, due to alternative splicing, in which the HSF2a isoform is predominantly expressed in adult tissue, while the HSF2b isoform is predominantly expressed in embryonic tissue.

During mitosis, the inducible Hsp70 promoter remains uncondensed. This is due to its binding to HSF2, which recruits protein phosphatase 2A and interacts with the CAP-G subunit of condensin, which contributes to the effective dephosphorylation and inactivation of condensin complexes in the immediate vicinity, thereby preventing condensation at this site. HSF2 also binds to HSE elements in the promoters of other heat shock genes, such as Hsp90 and Hsp27, as well as the c-Fos proto-oncogene. This indicates that HSF2 is important for the expression of HSE-containing genes [26].

Additionally, it is recognized that HSF2 can assemble HSF1 to form heterotrimers. Upon exposure to a specific stimulus, HSF1 becomes activated, and HSF1–HSF2 heterotrimers are formed. Heat shock stress decreases HSF2 levels, limiting the formation of heterotrimers and reducing the availability of HSF2. It has been proposed that the HSF1–HSF2 heterotrimer serves as a mechanism that coordinates the activation of transcription in response to specific stimuli during the process of development [23].

#### 2.1.3. HSF3

Initially, HSF3 was identified in avian cells, and to date, there is no evidence of HSF3 presence in other organisms. HSF3 also responds to heat stress, but its activation threshold temperature is higher than that of HSF1. HSF3 binds to c-Myb, a transcription factor involved in cell proliferation and essential for G1/S transition of the cell cycle, leading to the parallel expression of HSP70. Thus, the HSF3/c-Myb interaction may be implicated in the cell cycle-dependent expression of HSP70. The HSF3/c-Myb association is disrupted by the direct binding of the tumor suppressor transcription factor p53 to HSF3, resulting in the inhibition of HSP70 expression [27,28].

#### 2.1.4. HSF4

In mammals, HSF4 is highly expressed in lung and brain tissues, while its expression is nearly absent in other organs. At least two isoforms, HSF4a and HSF4b, are produced through alternative RNA splicing. HSF4a acts as a repressor of constitutive heat shock gene expression, while hHSF4b functions as a transcriptional activator. Thus, the differential splicing of HSF4 mRNA results in the production of both a repressor and an activator of tissue-specific heat shock protein gene expression, although this phenomenon remains poorly understood to date [29,30].

### 2.2. The Regulation HSP90 by Cochaperones

The chaperone activity of HSP90 is entirely dependent on the binding and hydrolysis of ATP. The primary forms of HSP90 include HSP90α, HSP90β, GRP94, and TRAP-1, which share a high degree of similarity in the N-terminal ATP-binding region. HSP90 is composed of three domains: the N-terminal domain, the C-terminal domain, and the middle domain. The N-terminal domain, which contains the ATP-binding site, is highly conserved among HSP90 family members. The ATP-binding site in the N-terminal domain is crucial for the chaperone cycle of HSP90. The middle domain of HSP90 is essential for binding clients and ATP hydrolysis, which occurs only after the ATP-binding site in the N-terminal domain interacts with the middle domain. The C-terminal domain of HSP90 contains three key motifs: a calmodulin-binding site, an HSP90 homodimerization motif, and a nucleotide-binding site. The calmodulin binding site may regulate the conformation of HSP90 by disrupting the intramolecular interaction. The nucleotide-binding site in the CTD exhibits ligand specificity and serves as an allosteric regulator of N-terminal ATPase activity [31].

HSP90 has a multitude of co-chaperones, with over 50 described variants. In the HSP90 cycle, the initial phase of co-chaperone modulation involves regulating the ATPase activity of HSP90, assisting in the recruitment of client proteins, and performing other specialized functions. During the ATPase cycle of HSP90, co-chaperones play a crucial role in regulating the conformational changes of HSP90 [31].

Numerous co-chaperones can modulate HSP90 ATPase activity. Aha1, a novel co-chaperone, was discovered and demonstrated to be required for HSP90 activation. Aha1 directly binds to HSP90, enhancing its weak intrinsic ATPase activity. The N-terminal domain of Aha1 interacts with the HSP90 middle domain, while the C-terminal domain dynamically binds to the HSP90 N-terminal domain. These interactions facilitate the dimerization of the HSP90 N-terminus, significantly accelerating its ATPase activity and shifting its equilibrium toward the closed conformation of HSP90. The activity of Aha1 contributes to the folding and maturation of client proteins. The reduction in Aha1 activity results in a decrease in HSP90 ATPase activity, which affects the folding of cystic fibrosis transmembrane conductance regulator (CFTR) [31].

Ids2 is a co-chaperone that works with HSP90 to enhance its chaperone activity. The interactions between the middle region of Ids2 and the NTD of HSP90 stimulate the ATPase activity of HSP90, which in turn recruits the client protein Atp3 to the HSP90 folding system. Ids2 is a mitochondria-dominant HSP90 co-chaperone and is important for mitochondrial function [31].

The co-chaperone p23 is crucial for suppressing of HSP90 ATPase. It possesses a unique domain known as the cysteine- and histidine-rich domain (CHORD). This domain interacts with HSP90 MD, specifically affecting the NTD-MD arrangement of HSP90 in the closed state. The interactions between p23 and HSP90 result in a 50% decrease in the ATPase rate. Alanyl-tRNA synthetase domain-containing 1 (Aarsd1), a novel co-chaperone of HSP90, shares similarities with p23in its ability to inhibit HSP90 ATPase activity [31].

The p23 and its *S. cerevisiae* homolog Sba1 tend to associate with HSP90 in the presence of ATP, which helps maintain the state necessary for activation of HSP90 client proteins. This regulatory feature of p23/Sba1 enhances the efficiency of the ATPase-dependent HSP90 cycle. The structure consists of a dimer of HSP90, with p23/Sba1 molecules symmetrically positioned on both sides. Consequently, the binding of p23/Sba1 can stabilize the ATP-bound conformation of HSP90 [31].

CDC37 is a crucial co-chaperone of HSP90, playing a vital role in regulating the HSP90 chaperone cycle. The functional dissection of CDC37 revealed a kinase-binding region at the N-terminus, while the rest of the protein interacted with HSP90. The structure of CDC37 revealed how it halts the HSP90 chaperone cycle by trapping the “lid” of the nucleotide binding site in an open conformation, preventing the “jaws” of the HSP90 molecular clamp from closing, thus facilitating the loading of client proteins [31].

The E3 ubiquitin ligase component cereblon (CRBN) is a conserved regulator, and CRBN-based proteolysis-targeting chimeras (PROTACs) have already been developed as potential evolutionary therapeutic agents. CRBN, as an HSP90 co-chaperone, specifically binds with the MD of the ATP-bound and closed states of the HSP90 dimer. This binding inhibits HSP90 ATPase activity, counteracting the negative effect of Aha1 on client protein stability [31].

Additional novel co-chaperones that slow down the HSP90 chaperone cycle but do not completely halt it include folliculin-interacting protein 1 (FNIP1), tissue inhibitor of metalloproteinases-2 (TIMP2), and tuberous sclerosis complex 1 (TSC1), which interacts with HSP90 MD and assists in the folding of related clients [31].

## 3. The Role of Other Transcription Factors in Modulating HSP Gene Expression via STAT and NF-IL6 Pathways

The STATs are a group of cytoplasmic proteins that act as transcription factors, mediating intracellular signaling initiated by cytokine receptors and transmitted to the nucleus. The STATs are activated by the phosphorylation of conserved tyrosine and serine residues in their C-terminal domains, which is facilitated by the janus kinase (JAKs) and MAP kinase families, respectively. This allows STATs to form dimers and translocate to the nucleus, where they regulate gene expression. Interferon-γ is a potent activator of STAT1, while interleukin-6 (IL-6), a leukemia inhibitory factor (LIF), and CT-1 primarily activate STAT3 [25].

It is known that the cytokine IL-6 stimulates two different signaling pathways, which leads to the activation of two different classes of cellular transcription factors. Studies have shown that many IL-6-induced genes contain binding sites to a transcription factor called IL-6 nuclear factor (NF-IL6). Subsequently, another representative of the NF-IL family, NF-ILß (or C/EBPδ), was identified, which can form heterodimers with NF-IL6, which leads to a synergistic the transcriptional effect. After exposure to IL-6 cells, NF-IL6 is phosphorylated, which leads to its increased ability to stimulate transcription, while NF-IL6b is synthesized de novo. The second pathway that is stimulated by IL-6 is the JAK/STAT3 signaling pathway.

It is widely acknowledged that NF-IL6/NF-IL6β and STAT3 signaling pathways enable IL-6 to trigger the activation of two different sets of genes, each responding to a specific pathways. Consequently, the genes responsible for the production of acute-phase proteins of Class 1 (such as α1-acid glycoprotein, haptoglobin, C-reactive protein, and serum amyloid) contain response elements to the factors NF-IL6 and NF-IL6b. These factors are involved in the activation of these genes following exposure to IL-6. Additionally, these genes are also stimulated by exposure to IL-1 cells, which activate NF-IL6/NF-IL6b without affecting STAT3. The genes responsible for the production of acute-phase proteins of Class 2 (such as fibrinogen, thiostatin, and a2-microglobulin) are not induced by IL-1 and do not contain binding sites to NF-IL6/NF-IL6b. Instead, these genes contain binding sites for STAT3, which is responsible for the activation of these genes in response to exposure to IL-6 [25].

IL-6 can induce an increased expression of Hsp90 in various cell types. It has been shown that the promoter of the HSP90β gene reacts to IL-6 and can also be activated by NF-IL6 or NF-IL6b. Moreover, a short region of the promoter containing the NF-IL6 binding site was necessary for the activation of both IL-6 and NF-IL6 promoters. This promoter region can provide sensitivity to both IL-6 and overexpression of NF-IL6 on a heterologous promoter. These data indicate that the Hsp90 gene is activated by IL-6 via the NFIL6/NF-IL6b pathway [32].

It is noteworthy that the short region of the promoter also encompasses binding sites for STAT3. The HSP90 promoter can be activated by this factor as well. Furthermore, overexpression of NF-IL6 and STAT3 produces a synergistic effect on the HSP90 promoter. Both of these signaling pathways appear to be essential for the activation of the HSP90 promoter through IL-6. However, these two pathways exert opposing effects on the regulation of the HSP90 promoter caused by heat shock: STAT3 reduces the stimulating effect of heat shock, while NFIL6 enhances it. When heat shock and IL-6 are combined, the activation of HSP90 is only weakly induced compared to when they are applied individually, indicating that the inhibitory influence of STAT3 on HSF prevails in this context. Conversely, IL-1, which only activates the NF-IL6 pathway, when combined with heat shock, results in a robust activation of Hsp90 [33].

Exposure to IFN-γ increases the levels of Hsp70 and Hsp90, and also enhances the activity of Hsp70 and Hsp90β promoters, and these effects depend on the activation of the STAT1 transcription factor IFN-γ. IFN-γ modulates the induction of Hsp through the STAT1-dependent pathway. The effect of IFN-γ/STAT1 was mediated by the same short region of Hsp70/Hsp90 promoters, which mediates the effects of NF-IL6 and STAT3 and can bind STAT1. This region also contains the binding site of the stress-activated transcription factor HSF1. STAT1 and HSF1 interact with each other through protein–protein interaction and cause strong transcription activation, while STAT3 and HSF1 do not interact directly.

Such protein–protein interactions and the binding of various transcription factors activated by stress and cytokines to the short promoter regions of the HSP90 and HSP70 genes likely play a crucial role in the activation of HSP genes by non-stress stimuli and in integrating these responses with the stress response of these genes. Moreover, STAT1 can interact with p53, and both these factors are capable of modulating the effects of HSF1 on HSP expression. Thus, various interactions of HSF1 with its partners can influence the HSF1-mediated transcriptional regulation of HSP proteins [34].

**Figure 1:**
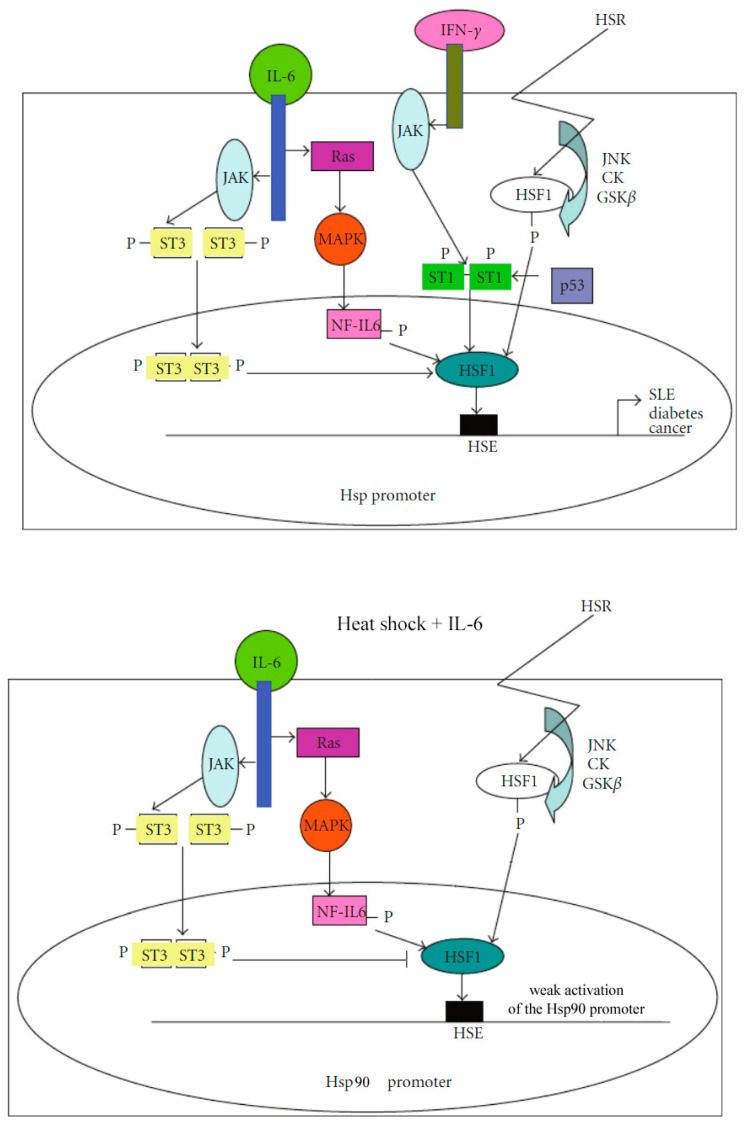
Signaling pathways activated by STAT1, STAT3, NF-IL6, and p53, and the heat shock response (HSR) through HSF1, which binds to the heat shock element (HSE) and modulates the transcription of heat shock proteins, the dysregulation of which is associated with various pathological diseases (modified from Stephanou A, Latchman DS, 2011 [25]).

A generalized schematic of the signaling pathways modulating HSP protein expression is presented in Figure 1.

## 4. Levels of Molecular Chaperones in Normal and Pathological Conditions

The chaperone system in an organism is comprised of molecular chaperones, co-chaperone factors, co-chaperones, and receptor chaperones. It is found throughout the body but has distinct features for each type of cell and tissue. Chaperones are cytoprotective, meaning they are involved in maintaining and adjusting protein homeostasis in health and disease. Cancer, neurodegenerative, inflammatory, and other diseases lead to perturbations of protein homeostasis and are often even promoted by them. Furthermore, some chaperones potentiate tumor growth, proliferation, and metastasizing. For example, HSP90 is the master regulator of the PI3K-Akt-NF-kB axis that promotes tumor cell proliferation and metastasizing [35]. In normal and pathological conditions, the level of molecular chaperones may be different, which affects the course of the disease.

Furthermore, genetic alterations, such as mutations in molecular chaperone genes and dysregulation of their expression levels, may contribute to the development of diseases.

### 4.1. Cardiovascular Diseases

The heart contracts throughout the life of mammals, and to do so, cardiomyocytes need to contract constantly using energy provided by mitochondria. They rely on a complex contractile apparatus comprised of transmembrane sarcomeric proteins to accomplish this. The proper folding and assembly of these sarcomeric proteins and numerous other proteins involved in ATP and Ca^2+^ exchange are crucial for maintaining normal cardiac function. When proteins are misfolded, they can acquire new functions by establishing new or altered protein–protein interactions, leading to a cascade of secondary effects, such as the formation of aggregates that sequester proteins from both regulatory and structural roles [36,37]. Low levels of molecular chaperones inside cells are unable to correct misfolded proteins, which can lead to cardiac disease. During the development of heart hypertrophy, cardiac failure, and ischemic/reperfusion injury, there is an overall increase in the number of chaperones, co-chaperones, and transcription factors that regulate them as a protective mechanism [38].

Additionally, intracellular HSPs decrease in acute ischaemic heart diseases. At the same time, extracellular HSP70, HSP90, and BAG-3 have been shown to increase in patients with heart failure and chronic diseases (e.g., hypertension) and elevated levels of circulating heat, and HSP70 in peripheral and renal vascular diseases has been reported [39,40].

### 4.2. Neurodegeneration Diseases

Some neurodegenerative diseases are often referred to as conformational disorders, as their occurrence and progression depend on the pathological transformation of proteins with altered structures. This transformation results in a change in the structure of amyloidogenic proteins and the formation of aggregates from these proteins with abnormal conformations. Both these processes are influenced by various intracellular proteins termed chaperones. These chaperones are responsible for correct protein folding and prevent protein aggregation. They can also destroy already formed protein aggregates. However, the synthesis of these chaperones decreases with age in most cells. When the overall levels of chaperones become inadequate, newly synthesized (or generated through proteolysis) disease proteins undergo an intramolecular structural change during the early stages of aggregation, forming a β-structured monomer with exposed hydrophobic regions. Due to these hydrophobic regions, the monomer can accumulate in ring-like or spherical intermediates, either on or off the pathway for fibril formation. These intermediates can exert toxicity by creating membrane pores or by inactivating essential cellular factors [41,42,43,44,45].

### 4.3. Cancer Diseases

Cancer cells, in contrast to normal cells that are undergoing normal aging, undergo a de novo increase in heat shock protein (HSP) levels. This increase in HSP expression has been attributed to the increased folding demand in cancer cells or to the evolution of new mechanisms for inducing the heat shock response in these rapidly adapting cells [45]. High expression of HSP70 and HSP90 has been linked to enhanced tumor development and tumor aggressiveness. These proteins are critical regulators of the unfolded protein response (UPR), mitochondrial bioenergetics, lipid metabolism, and apoptosis, as well as innate and adaptive immune responses [35,46]. Inhibition of the central chaperone, HSP70, in cancer cells leads to a compensatory upregulation of other chaperones in the network. Both low and high levels of the non-essential factor (NEF) HSP110 have an inhibitory effect on HSP70 [47,48].

### 4.4. Autoimmune and Inflammatory Diseases

Heat shock proteins (HSPs) play a unique role in immunology, acting as both protective chaperones and mediators of disease. These proteins demonstrate remarkable functional flexibility, participating in both cellular homeostasis and pathological processes through specific mechanisms [49].

The intracellular heat shock protein (HSP) network, comprising HSP60, HSP70, and HSP90 as well as endoplasmic reticulum-resident HSP90B1/gp96, extends beyond its classical protein-folding functions and actively shapes immune responses. In systemic lupus erythematosus (SLE), elevated HSP90 expression within peripheral blood mononuclear cells reflects disease activity, suggesting its involvement in breaking immune tolerance [49]. Similarly, the upregulation of HSP60 and cytosolic HSPs in type 1 diabetes facilitates β-cell antigen presentation through both conventional and non-conventional pathways, ultimately leading to the autoimmune destruction of pancreatic islets [49].

The transition of heat shock proteins (HSPs) to extracellular compartments is a critical step in the pathogenesis of disease. Cellular stressors, such as apoptosis and viral infection, especially those that induce endoplasmic reticulum (ER) remodeling, trigger the release of HSPs [50]. Once outside the cell, these proteins play dual immunogenic roles. They establish pro-inflammatory microenvironments by signaling danger and simultaneously chaperone autoantigens to antigen-presenting cells (APCs). This dual functionality explains the elevated levels of anti-HSP autoantibodies observed in multiple autoimmune conditions, creating the perfect conditions for autoreactive T-cell activation [49,50].

The clinical significance of HSP dysregulation goes beyond classical autoimmune diseases. Infectious challenges, such as hepatitis C virus (HCV) infection, trigger a global increase in HSP as part of the body’s stress response [51]. Bacterial pathogens developed mechanisms to use HSP pathways to evade the immune system [52,53]. Metabolic disorders, including mucopolysaccharide diseases, demonstrate chaperone deficiency, with significantly reduced levels of HSP70 and HSP40 [54]. Neurological conditions, such as distal motor neuropathy, show selective depletion of HSP90 [55]. Quantitative profiling, as shown in Table 1, reveals distinct pathological patterns of HSP dysfunction across disease states. The extracellular concentration of specific HSPs is altered in each condition, reflecting their clinical and pathological involvement.

The emerging understanding of extracellular heat shock proteins (HSPs) as dual-function molecules that can both promote and suppress immune responses, depending on the context, has important implications for therapy. In particular, in the field of oncology, HSPs exhibit paradoxical abilities to enhance tumor antigen presentation, while also potentially fostering an immunosuppressive microenvironment. This knowledge opens up new possibilities for targeted immunotherapy development [49,50].

Moreover, chaperones are proteins, and as such, can be affected by mutations. At least 15 pathologic conditions have been reported where a gene encoding for a chaperone or a molecule with structural features similar to known chaperones is mutated or missing. These conditions, known as genetic chaperoneopathies, are characterized by abnormalities in nervous, muscular, or other tissues and may be considered to be inborn errors in development [73].

## 5. Models of HSPs Expression Modulation

### 5.1. Physical Models

Heat shock proteins protect cells from proteolytic stress, and thus the simplest models for modulating HSP expression levels in cultured cell lines are based on the application of physical stimuli to cells. Thus, increases or decreases in temperature, radiation, reactive oxygen species (ROS) production, hypoxia, mechanical damage, and cellular starvation generally lead to an increase in the levels of HSP60, HSP70, and HSP90 [2,3,4,5,6]. Moreover, exposure to heavy metals, such as cadmium and arsenite, as well as amino acid analogues and sulfhydryl-reactive agents, has also been shown to induce the expression of HSPs [5]. These diverse stressors disrupt protein homeostasis by promoting the unfolding, misfolding, or aggregation of proteins, which activates the heat shock response mediated by heat shock factors binding to heat shock elements in target gene promoters.

### 5.2. Pharmacological Models

Chemical compounds can interact with heat shock proteins, either increasing or decreasing their expression levels or activity. As summarized in Table 2, Table 3 and Table 4, the most well-known inhibitors and inducers of heat shock proteins are classified by their predominant mode of action.

Interestingly, some HSP90 inhibitors lead to the overexpression of HSP72 (the inducible form of HSP70) and many other heat shock proteins through the release of HSF1: the HSF1 transcription factor is held in an inactive state in a complex with HSP90, and when HSP90 activity is inhibited, it is released [113]. Moreover, chemotherapy drugs (such as betulinic acid or cisplatin) in combination with physical stimuli (e.g., electric fields) can increase the expression of HSP27 and HSP70 [114].

### 5.3. Genetic Models

Artificially increasing or decreasing the expression levels of HSP proteins can be achieved using genetic engineering constructs. To date, molecular cloning offers numerous systems for modulating the expression of various genes.

The knock-in (KI) system, or gene “insertion” system, involves the insertion of a specific informational DNA sequence into a particular locus using vectors, or the equivalent replacement of one gene locus with another. This gene insertion technique can lead to a permanent increase in the expression of a specific gene [115].

The knock-down (KD) system implies a decrease in the expression of one or more genes. Small RNAs, such as siRNA, shRNA, and miRNA, are most commonly used. Although these small RNA variants have slightly different mechanisms of action, they all can suppress the expression of target genes through RNA interference. Using vectors, small RNAs are introduced into the cell, where they bind to complementary DNA or RNA molecules. Subsequently, these complexes undergo degradation, and gene expression is halted at the site of RNA interference. As a result, gene expression is reduced. The extent of reduction can vary and depends on the locus where the interference occurs [116].

The knock-out (KO) system implies the complete and irreversible cessation of the expression of a specific gene due to alterations in genomic DNA. Unlike knock-down methods, gene knock-out techniques damage specific genes, rendering them nonfunctional. If cells or model organisms survive the knock-out, they will never be able to revert to expressing a functional gene product. Gene knock-out does not necessarily mean that the entire gene must be physically removed or excised from genome. It is sufficient to introduce a frameshift mutation, leading to the appearance of a stop codon. Typically, such as a mutation is introduced near the 5’ end of the gene (close to the transcription start site), transcription stops after the stop codon, and the gene becomes knocked out. The most commonly used methods for knock-out include homologous recombination, CRISPR-Cas9, and TALENs [117].

## 6. Mouse Transgenic Models

Currently, genetic models for modulating the expression levels of specific proteins can be applied not only at the cellular level, but also at the organismal level. For this purpose, transgenic laboratory animals, particularly mice, are widely employed as physiologically relevant systems to elucidate the mechanistic roles of heat shock proteins (HSPs), their functional interplay in proteostasis networks, and their participation in stress-responsive signaling pathways.

Despite the complexity and time-intensive nature of transgenesis, these methodologies have become indispensable tools for both fundamental research and translational applications. Transgenic animal models enable systematic investigation of HSP-mediated molecular cascades under pathological conditions, thereby advancing our understanding of disease pathogenesis and facilitating the development of targeted therapeutic strategies. For instance, genetically modified murine models are routinely utilized to decipher the molecular regulation of gene expression during mammalian development, establish physiologically congruent systems for modeling human diseases, and characterize the tissue-specific functions of HSPs in maintaining cellular homeostasis.

Furthermore, transgenic organisms serve as bioreactors for producing medically relevant human proteins, including pharmaceutical-grade HSPs with potential clinical applications.

**Figure 2:**
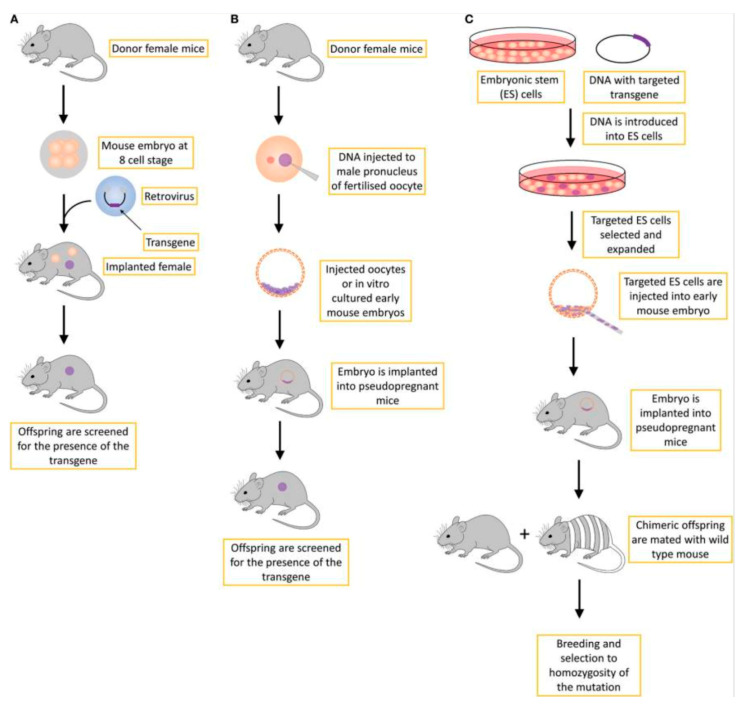
Strategies for generating transgenic animals (Lampreht Tratar et al., 2018 [118]): (**A**) retroviral approach, where embryo cells are infected using retroviral vectors at early development stages before implantation into a recipient female (rarely used); (**B**) microinjection method, the standard transgenic approach, where DNA is inserted into the genome in a non-specific manner; and (**C**) injection of genetically modified embryonic stem cells into a pre-implantation embryo at early development states. This is a gene-targeting transgenic approach, typically used to create knock-out transgenic mice with constitutive loss-of-function mutations.

Transgenic technologies for introducing foreign genes into fertilized eggs were developed and refined using laboratory mice, and since the early 1980s, hundreds of genes have been introduced into various mouse strains. Thus, to date, there are three main strategies for generating transgenic animals (Figure 2) [118]:

In the future, it will be possible to create mouse models for modulating heat shock proteins, which will aid in studying their roles in various diseases. However, it is important to note that due to alterations in the expression levels of housekeeping proteins, transgenic mouse models may prove to be non-viable.

### 6.1. Overexpression/Underexpression (Knock-In and Knock-Down)

In knock-in models, overexpression of a specific gene or insertion of an additional gene (e.g., a reporter gene) occurs. Most commonly, an additional copy of the gene of interest or several of its initial exons are inserted. Using homologous recombination, a more predictable and stable knock-in model can be achieved. The most frequently used site is the Rosa26 locus, as it does not contain any essential genes and ensures stable and predictable transgene expression in various types [119,120].

Gain-of-function studies are often used to investigate oncogenes in mouse models. Knock-in models overexpressing an oncogene can be employed to study how the oncogene drives carcinogenesis in vivo [118].

In knock-down models, the expression of a specific gene is reduced by approximately 70%, but its complete loss of function is not achieved. RNA interference is the most common method for achieving gene knockdown in such models. Double-stranded interfering RNA (siRNA) is introduced into the cytoplasm of embryonic stem cells, often leading to significant disruption of gene function in the developing mouse embryo. Since this process is rapid and inexpensive, it provides researchers with a unique and simple method for genetic functional analysis [121].

### 6.2. Suppression of Expression (Knock-Out, CRISPR-Cas9, and Humanized Mice)

Knock-out mouse models are frequently created for studying gene functions, investigating pathogenic mechanisms, and developing drugs.

Knock-out mouse models are genetically modified in such a way that the function of one or more specific genes is disrupted or inactivated. These genomic alterations result in changes in the phenotype of the model animals.

The first strategy for generating knock-out models is homologous recombination. In this strategy, an artificial DNA fragment, which has an identical or homologous sequence to the gene of interest, is introduced into the nucleus of embryonic stem cells. This homologous sequence flanks the DNA sequence of the existing gene both upstream and downstream of its chromosomal location. The cell’s intrinsic nuclear mechanism automatically recognizes the identical sequence regions and replaces the existing gene or its portion with the artificial DNA fragment. Since the artificial DNA is unactive and contains only a genetic marker or “reporter gene” designed for tracking purposes, the replacement eliminates the function of the existing gene [122].

Another popular method for generating knock-out models utilizes TALEN genome editing technologies. Using plasmids, a sequence recognizable and cleavable by TALEN is introduced into the target DNA sequence of the mouse. Non-homologous end joining is then employed to repair the damage caused by the cleavage. Repair of the damage is crucial, as the cell’s repair attempts can lead to irreversible damage and deletion errors, rendering knock-down mice unsuitable for use. The use of TALEN can result in the creation of both knock-out and knock-down models [123].

Currently, the CRISPR/Cas9 technique is also widely used for the rapid and stable generation of knock-out and knock-in mouse models.

Thanks to cutting-edge technologies, it is possible to achieve both precise point mutations and the insertion of large fragments. CRISPR utilizes small guide RNAs (sgRNAs) that direct the Cas9 nuclease to a specified genomic site using a ~20 nucleotide complementary sequence. Then, Cas9 creates a double-strand break at the target loci, which is either repaired in an error-prone manner to produce a frameshift mutation (knock-out) or restored through recombination with donor DNA containing homologous ends for integration at the break site (knock-in) [124].

Another scenario involves replacing the target gene with cDNA or a reporter gene, where knock-out occurs simultaneously with knock-in. Humanized mouse models can also be created by replacing the mouse gene with its human ortholog. In this case, part or all of the mouse gene sequence is replaced by its human counterpart, and the human gene is expressed under the control of the mouse regulatory sequence [125].

There are limitations to using knock-out mice: approximately 15% of such models are lethal during embryonic development, meaning that genetically modified embryos cannot develop properly and reach adulthood [126].

Knock-in and knock-out of HSP70 mice models are widely used in cardiac dysfunction and aging research. Myocardial recovery after ischemia [127,128,129] and skeletal muscle dysfunction in aging [130] were studied in transgenic mice overexpressing HSP70. Knock-out of HSPa1a and HSPa1b genes in mice were generated for the study dysfunctional cardiomyocytes and functional and cellular damage by ischemia/reperfusion [131].

There is also a humanized transgenic mouse strain which expresses the modified high-quality, uniform, and reproducible human HSP70 in milk and which is easy to isolate using ATP columns [132].

### 6.3. Regulated Expression

Constitutive knock-in or knock-out of a gene can lead to lethality, sterility, and developmental defects. Therefore, it is necessary to regulate the spatial and temporal control of gene expression to overcome these limitations in such models. Conditional knock-in expression models can be created using tissue-specific promoters, by inserting strong translational and transcriptional termination sequences, or by utilizing the tetracycline-inducible expression system [118].

### 6.4. Tissue-Specific Promoters

A tissue-specific promoter is a promoter that is active only in certain cell types. Using a tissue-specific promoter in an expression cassette when creating genetic models can limit unwanted transgene expression and promote stable transgene expression in the desired location (organ or tissue). However, when constructing a vector with the gene of interest under a tissue-specific promoter, several factors must be considered. Ideally, the elements of the natural promoter region required to achieve the desired level of gene expression while maintaining tissue specificity should be known. Additionally, it is important to understand whether interactions occur between the promoter region and the rest of the vector genome, as these may affect the activity and specificity of the promoter [133].

### 6.5. Cre-loxP and FLP-FRT Systems

The fundamental elements of this technology are bacterial Cre enzymes and yeast FLP enzymes. These enzymes act as specific recombinases, catalyzing the recombination between specific 34-bp loxP and FRT sites. A strong translational and transcriptional termination (STOP) sequence is inserted into the genome. This sequence is flanked by either loxP or FRT sites and positioned between the promoter sequence and the gene of interest. When Cre or FLP proteins become active, they induce homologous recombination between the loxP and FRT sites. These sites are adjacent and are oriented in the same direction at the gene of interest, resulting in its subsequent deletion after recombination occurs in the lateral regions of the genetic sequence [134].

In order to achieve spatial control, it is necessary to first obtain mice that express Cre or FLP recombinases regulated by a promoter that is specific to a particular tissue or can be induced. These mice are then crossed with target mice carrying a gene of interest that is flanked by either loxP or FRT sites. This allows for the conditional inactivation of the target gene in specific tissues, cellular types, or during specific stages of development [135].

Tissue-specific models can also be obtained using virus-inducible vectors, which are introduced locally or into specific sites via direct injection into cells, thereby delivering Cre or FLP enzymes to the tissues or target cells. Both adenoviral and lentiviral vectors can be used for this purpose [136].

In the case of tetracycline-based systems, a transactivator and an effector are used. The tetracycline-controlled transactivator protein (tTA) binds to the tetracycline operator (tetO), which regulates the expression of the gene of interest. When tetracycline is added to an animal’s drinking water, it binds to tTA, preventing it from binding to tetO and blocking gene transcription. This is known as the Tet-off system, where gene expression is suppressed in the presence of tetracycline. The reverse tetracycline-controlled transactivator protein (rtTA), on the other hand, only binds to tetO when it is bound to tetracycline. This allows for the initiation of gene expression when tetracycline is present in the animal’s body. However, one disadvantage of this system is that rtTA can leak, potentially compromising the desired regulation of transgene expression. RTTA retains some affinity for tetO sequences, even in the absence of tetracycline. This leads to the undesirable transcription of target genes [137].

### 6.6. Tamoxifen-Inducible Expression System

In the tamoxifen-induced system, the gene of interest fuses with the mutated ligand-binding domain of the human estrogen receptor (CRE-ER(T)), which is specifically activated by tamoxifen. When the active metabolite of tamoxifen, 4-hydroxytamoxifen, is present, the ER fusion protein remains in the nucleus. However, when 4-hydroxytamoxifen is absent, the fusion protein is removed from the nucleus. Upon binding to tamoxifen, the fused ER protein is transported back into the nucleus, allowing the gene of interest to be expressed. Consequently, the temporal expression of the gene of interest can be controlled by administering tamoxifen to the animals or withholding it [138].

Tetracycline- and tamoxifen-inducible systems can be used to create temporal Cre or FLP models [137,138].

A mouse strain was developed using the heat shock protein 70 (HSP70) promoter, allowing for the activation of CreERT2 in response to physical stimuli. This was achieved to investigate the processes of physical therapy, tissue repair, and regeneration, as well as to trace lineage and modulate gene expression in cells within the stimulated tissue-specific time points. This mouse model can reveal which cells are impacted by physical stimuli [139].

Although there are various transgenic mouse models associated with altered HSP70 expression, they are not often used in the study of cancer or neurodegeneration. Heat shock proteins play an important role in oncological diseases; therefore, the creation of various transgenic animal models that modulate the expression of various proteins of the heat shock protein family will become a convenient tool in the study of such socially significant diseases such as cancer and neurodegeneration.

### 6.7. PDX Mice Models and Their Use

Patient-derived xenograft (PDX) is a mouse model created by implanting a fragment of a human tumor into an immunodeficient or humanized mouse strain. Such models will exhibit the protein expression profile, including HSPs, characteristic of the specific patient and replicate the behavior of that patient’s cancer as accurately as possible.

Currently, PDX mice are widely used in cancer research to identify potential new treatments, discover new therapeutic targets, and understand the causes and biology of oncological diseases [140].

One of the next steps in advancing PDX mouse models is to endow the mice with a functional human immune system, enabling their use for testing immunotherapeutic drugs. The most common approach to achieving this involves transplanting human stem cells, which generate blood and immune cells, into the mice [141].

## 7. Conclusions

In conclusion, proteins from the heat shock proteins family (HSPs) are important in maintaining cellular homestasis, and changes in their expression levels can have different effects on the progression and pathology of a particular disease. Therefore, it is important to choose the right model of modulation of the expression of HSPs, taking into account all the limitations and advantages (Table 5). It is worth noting that changes in the expression level of heat shock proteins determine the pathogenesis of various diseases. Thus, neurodegenerative diseases (e.g., Parkinson’s disease, Alzheimer’s disease, etc.) can be associated with a reduced level of chaperones, while oncological processes are associated with a high level of HSPs. In this regard, modulation of the HSP level in the body using both various physical factors (for example, hyperthermia) and chemical molecules (either inducers or inhibitors) should be based on the contribution of chaperones to the pathogenesis of the disease: presumably, for neurodegenerative diseases, an approach based on a compensatory increase in HSP can be an optimal tactic, while suppression of HSP in oncological diseases should take place. Considering that molecular chaperones form close functional connections and suppression of one chaperone compensatorily leads to an increase in the activity and expression of other chaperone molecules, it is advisable to use several inhibitors aimed at different nodes of the chaperone network (for example, a combination of HSP70 and HSP90 inhibitors) [142]. Moreover, considering that many methods of antitumor therapy, such as radiochemotherapy, promote an increase in the response expression of HSP in cancer cells, it is advisable to combine these methods with chaperone inhibitors.

Given the advantages and disadvantages of each HSP modulation model, it is best to use several models in research, which will allow for a more correct understanding of the pathogenesis of the disease under study and identify new targets for treatment.

## Figures and Tables

**Table 1:** Systemic alterations in heat shock protein concentrations across pathological conditions: comparative analysis of HSP70, HSP90, and HSP27 levels in serum/tissues from healthy and diseased cohorts.

Pathological Conditions	Disease Conditions	Physiological Levels of Biomarker in Non-Disease Condition (ng/mL)	Levels of Biomarker in Disease Condition (ng/mL)	Source
HSP90 (serum)				
Cancer diseases	Skin cancer (melanoma)	27.07	49.76	[56]
Cardiovascular diseases	Acute pulmonary embolism	11.54 ± 3.43	15.30 ± 7.11	[57]
HSP90α (serum)				
Cardiovascular diseases	Hypertension	5.56 ± 3.00	11.47 ± 7.81	[58]
HSP90β (serum)				
Cancer diseases	Head and neck squamous cell carcinoma (HNSCC)	23.5 ± 3.8	65.6 ± 13.08	[59]
HSP27 (serum)				
Cancer diseases	Salivary gland tumor	0.602 ± 0.575	malignant salivary gland tumor: 3.956 ± 3.830 benign salivary gland tumor: 0.752 ± 0.485	[60]
	Ovarian cancer	13.65 ± 11.62	50.17 ± 57.59	[61]
Cardiovascular diseases	Acute pulmonary embolism	7.44 ± 1.94	9.95 ± 4.09	[57]
HSP70 (serum)				
Cancer diseases	Pancreatic cancer	0.04	1.68 ± 0.083	[62]
	Breast cancer	Not reported	2.11–2.51	[63]
	Prostate cancer	0.3–0.8	0.5 ± 2.0	[64]
Cardiovascular diseases	Coronary artery disease	0.72	0.34	[65]
	Acute myocardial infarction	Not reported	0.26–0.76	[66]
	Chronic coronary syndrome	Not reported	0.55–1.63	[66]
	Vascular mild cognitive impairment	11.32	14.11	[67]
Neurodegeneration diseases	Alzheimer’s disease	11.32	10.16	[67]
	Mild cognitive impairment	11.32	10.16	[67]
	Multiple sclerosi	6.0	8.0	[68]
	Clinically isolated syndrome	6.0	8.2	[68]
	Relapsing–remitting multiple sclerosis	6.0	9.3	[68]
	Secondary progressive multiple sclerosis	6.0	7.5	[68]
	Primary progressive multiple sclerosi	6.0	5.3	[68]
	Non-inflammatory neurological diseases	6.0	7.3	[68]
Autoimmune and inflammatory diseases	Rheumatoid arthritis	0.12–0.42	high disease activity: 1.66 ± 0.75 low disease activity: 0.49 ± 0.1 moderate disease activity: 0.52 ± 0.12 remission phase: 0.48 ± 0.11	[69]
Other diseases	Psoriasis	2.57 ± 1.52	3.31 ± 1.92	[70]
	Other inflammatory neurological diseases	6.0	11	[68]
HSP70 (intracellular)				
Cancer diseases	Breast cancer	44.2	79.3	[71]
HSP70 (circulating)				
Cancer diseases	Not reported	Not reported	Cancer patients without chemotherapy: 783.2–802.7 Cancer patients who were treated with chemotherapy: 301.4–513.3	[72]
	Recurrent breast cancer	Not reported	512.4	[72]

**Table 2:** Inhibitors of heat shock proteins.

Name	Description	Action	Source
Geldanamycin	A benzoquinone compound, ansamycin derivative.	Inhibitor of HSP90 and the family of nuclear hormone receptors. Geldanamycin and related molecules inhibit the chaperone functions of HSP90 by competing for ATP binding.	[74,75,76]
Pifithrin-μ	A cell-permeable sulfonamide.	Potent inhibitor of HSP70. Interacts with the substrate-binding domain of the chaperone protein and disrupts its interaction with client proteins.	[77]
Gedunin	Pentacyclic triterpenoid.	Potent inhibitor of HSP90, induces degradation of HSP90-dependent client proteins.	[78]
Monorden (Radicicol)	Natural compound first isolated from the saprophytic fungus *Monosporium bonorden.*	Selective inhibitor of HSP90, interacts with the ATP-binding component, disrupting its chaperone function.	[74,79]
Macbecin I	Antibiotic from the benzoquinone ansamycin class. Natural compound, produced by bacteria of the species Micromonospora.	Stable inhibitor of HSP90 activity, interacts with the ATP-binding domain.	[80,81]
Heat Shock Protein Inhibitor I	Benzylidene lactam compound.	Interacts with the substrate-binding domain and prevents the synthesis of inducible heat shock proteins: HSP40, HSP70, HSP72, and HSP105.	[82,83]
Heat Shock Protein Inhibitor II	Metabolite of Heat Shock Protein Inhibitor I.	Interacts with the ATP-binding domain of heat shock proteins and prevents their synthesis, particularly HSP90.	[84]
PF-04929113	Mesylate	Selective and potent inhibitor of HSP70 and HSP90, targets the ATP-binding site.	[85,86]
VER–50589	Isoxazole compound.	Selective HSP90 inhibitor, interacts with the substrate-binding domain	[87]
VER–155008	Derived from adenosine.	Inhibitor of HSP70, binds to Hsc70 and Grp78, and is selective for HSP 90beta.	[88]
NSC 630668-R/1 MAL3-101 DMT3132 DMT3024 MAL2-11B 15-deoxyspergualin (DSG)	Dihydropyrimidine derivative.	Inhibitors of HSP70, interact with the ATP-binding domain.	[89,90,91,92,93,94,95,96]
Epigallocatechin-3-gallate (EGCG)	Natural compound, derived from green tea leaves.	Inhibitor of HSP90, inducer of HSP70. Interacts with the ATP-binding domain of HSP70. Suppresses GRP78.	[97]
Quercetin (3,3’,4’,5,7-pentahydroxyflavone)	Natural substance from the flavonoid group, first isolated from oak bark.	Inhibitor of HSP70.	[98]
Onalespib (AT13387)	Synthetic NTD ATP binding site competitor.	Inhibitor of HSP90.	[99,100]
Luminespib (NVP-AUY922)	Resorcinylic isoxazole amide.	A third-generation inhibitor of HSP90, upregulated HSP72, binds with high affinity to the HSP90 NTD ATP pocket.	[99,100]
HS-196 and HS-201	A near-infrared fluorescent molecule linked to the human HSP90i analog-SNX-5422 (HS-196). A theragnostic version of the HSP90-binding drug HS201 was developed by replacing the near IR molecule with the photosensitizing molecule verteporfin.	Selectively and competitively binds to upregulated HSP90.	[99,100]
Gamitrinib	TPP hexafluorophosphate.	Mitochondrial matrix inhibitor that links GA HSP-90 inhibitor 17-AAG to triphenyl phosphonium, which is an efficient mitochondrial import carrier-based mitochondrial matrix targeted HSP90.	[99,100]
Zelavespib (PU-H71)	Purine-based compound.	Inhibitor of Hsp90 with potential antineoplastic activity. Specifically inhibits active Hsp90, thereby inhibiting its chaperone function.	[99,100]
SNX-5422	Synthetic, novel, small molecule inhibitor.	Direct inhibitor of HSP90.	[99,100]
Ganetespib (STA-9090)	Second-generation, resorcinol derivative.	Inhibitor of Hsp90, that binds to the NTD ATP pocket of HSP90.	[99,100]
XL888	Bioavailable, small-molecule inhibitor.	Potent and orally active inhibitor of HSP90.	[99,100]
Pimitespib (TAS-116)	Novel, small-molecule inhibitor.	Selectively ATP-competitive inhibits cytosolic HSP90 isoforms α and β and not the endoplasmic reticulum and mitochondrial paralogs (GRP94 and TRAP1).	[99,100]
PEN-866	Miniaturized drug conjugate linked to SN-38, the active metabolite of irinotecan.	Inhibitor of Hsp90. Targets and is retained in the tumor due to its binding to HSP90.	[99,100]

**Table 3:** Inducers of heat shock proteins.

Name	Description	Action	**Source**
U-133	Water-soluble derivative of naphthoquinone echinochrome 1, isolated from the sea urchin Scaphechinus mirabilis.	Inducer of HSP70.	[101,102]
Aspirin	Acetylsalicylic acid.	Inducer of HSP70.	[103]
Myricetin	Flavonoid, plant polyphenol.	Increases intracellular levels of HSF-1 and HSP70.	[104]
Celastrol	Quinone methide isolated from the grape Tripterygium wilfordii.	Enhances HSF-1, which in turn leads to the induction of HSP70.	[105]

**Table 4:** Inducers and inhibitors of heat shock proteins.

Name	Description	Action	Source
Synthetic analogs and derivatives of geldanamycin: 17-AAG (Tanespimycin) 17-DMAG	Cyclic amides of aminocarboxylic acids.	Inhibitor of HSP90, inducer of HSP70.	[106,107,108]
J2	Heterocyclic organic compound.	Inhibitor of HSP27, induces abnormal dimer formation of HSP27 and suppresses the production of giant HSP27 polymers, thereby inhibiting its chaperone function.	[109]
NVP-AUY922 AT-13387 GGA (geranylgeranylacetone)	Resorcinases: radicicol-based compounds.	Inducer of HSP70, inhibitor of HSP90, interacts with the ATP-binding domain of HSP90.	[110,111,112]

**Table 5:** Advantages and disadvantages of different models of HSP expression modulation.

Models	Advantages	Disadvantages
Physical models	simple and cheap native response	only induction non-specific modulation
Pharmacological models	specific modulation induction and inhibition	absence of inhibitors and inducers to all proteins of the family HSP expensive
Genetic models	specific modulation of induction and inhibition induction and inhibition controlled modulation	expensive labor-intensive time consuming
Mouse transgenic models	specific modulation of induction and inhibition induction and inhibition modulation in space and time controlled modulation	expensive difficulty in obtaining labor-intensive time consuming possibility of lethal mutations
PDX mice models	full compliance with the patient’s protein profile personalized medicine	expensive difficulty in obtaining not controlled modulation

## Data Availability

No new data were created or analyzed in this study.

## Abbreviations

17-AAG	17-allylamino-17-demethoxy-geldanamycin
17-DMAG	17-Dimethylaminoethylamino-17-demethoxygeldanamycin
ATP	Adenosine triphosphate
Bip	Binding immunoglobulin protein
CK2	Casein kinase 2
DNA	Deoxyribonucleic acid
Grp75	75 kDa glucose-regulated protein
Grp78	78 kDa glucose-regulated protein
GSK3β	glycogen synthase kinase 3β
HSE	heat shock element
HSPs	heat shock proteins
HSR	heat shock response
IFN-γ	Interferon-gamma
IL-6	interleukin 6
JAKs	Janus kinases
JNK	c-jun N-terminal kinase
LIF	leukemia inhibitory factor
mRNA	messenger RNA
NF-IL6	nuclear factor IL-6
P53	Tumor protein P53
RNA	Ribonucleic acid
*siRNA*	Double-stranded interfering RNA
SIRT1	silent mating type information regulation 2 homolog
STAT	signal transducer and activator of transcription
TNF-α	tumor necrosis factor α
TRiC	T-complex protein ring complex
